# Enzymatic synthesis of tryptamine and its halogen derivatives selectively labeled with hydrogen isotopes

**DOI:** 10.1007/s10967-013-2816-0

**Published:** 2013-11-10

**Authors:** Sylwia Dragulska, Marianna Kańska

**Affiliations:** 1 Department of Chemistry, University of Warsaw, Pasteur Str. 1, 02-093 Warsaw, Poland; 2 Medical University of Warsaw, Żwirki i Wigury Str. 61, 02-091 Warsaw, Poland

**Keywords:** Decarboxylation, Deuterium, l-Phenylalanine decarboxylase, Tritium, Tryptamine derivatives, Tryptamine

## Abstract

Nine isotopomers of tryptamine and its halogen derivatives, labeled with deuterium, tritium in side chain, i.e., [(*1R*)-^2^H]-, [(*1R*)-^3^H]-, 5-F-[(*1R*)-^2^H]-, 5-F-[(*1R*)-^3^H]-, 5-Br-[(*1R*)-^2^H]-, double labeled [(*1R*)-^2^H/^3^H]-, 5-F-[(*1R*)-^2^H/^3^H]-, and ring labeled [4-^2^H]-, and [5-^2^H]-tryptamine, were obtained by enzymatic decarboxylation of l-Trp and its appropriate derivatives in deuteriated or tritiated media, respectively. Intermediates: [5′-^2^H]-l-Trp used for further decarboxylation was synthesized by enzymatic coupling of [5-^2^H]-indole with *S*-methyl-l-cysteine, and [4′-^2^H]-l-Trp was obtained by isotope exchange ^1^H/^2^H of the authentic l-Trp dissolved in heavy water induced by UV-irradiation. Doubly labeled [(*1R*)-^2^H/^3^H]- and 5-F-[(*1R*)-^2^H/^3^H]-tryptamine were obtain by decarboxylation of l-Trp or [5′-F]-l-Trp carried out in ^2^H^3^HO incubation medium.

## Introduction

Tryptamine, a biogenic amine is also the backbone for a group of compounds known collectively as tryptamines. This group includes many biologically active, natural or synthetic compounds, including neurotransmitters, e.g., serotonin, melatonin [1, 2] and psychedelic drugs such as DMT (*N*,*N*-dimethyltryptamine) [3, 4], and psilocybin. Diversity of tryptamine derivatives due to the large number of substituents in the different positions of the indole ring and the aliphatic chain, therefore, this class of compounds has been widely used in medicinal chemistry for production the psychoactive drugs. The tryptamines have a multiple applications: they regulate many biologically processes, affect the nervous system (serotonin) [5], and are involved in the sleep-wake cycle (melatonin) [2].

**Fig. 1:**
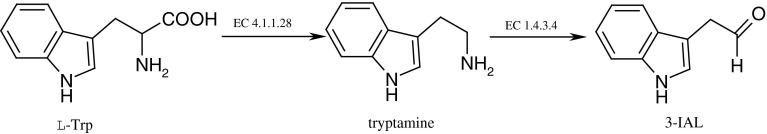
The metabolic pathway of l-Trp to 3-indolacetaldehyde, 3-IAL

In mammals brain tryptamine, acting as an endogenous neurotransmitter, is synthesized by the enzymatic decarboxylation of l-tryptophan (l-Trp) catalyzed by aromatic l-amino acid decarboxylase [AADC (*EC 4.1.1.28*)], and is metabolized to 3-indolcetaldehyde (3-IAL) by oxidative deamination catalyzed by the enzyme monoamine oxidase (*MAO, EC 1.4.3.4*) [6] (Fig. 1).

In the plants and mammalian the bioamines, products of enzymatic decarboxylation of amino acids play a role of precursors of a wide range of alkaloids and other biologically active compounds [7]. The enzyme aromatic l-ADDC (*EC 4.1.1.28*), involved in production of tryptamine [8] is also an effective catalyst in the decarboxylation of various aromatic l-amino acids 3,4-dihydroxy-l-phenylalanine (l-DOPA), l-tryptophan, 5′-hydroxy-l-tryptophan, phenyl-l-alanine, l-tyrosine, and l-histidine [9]. This pyridoxal phosphate (PLP) containing enzyme is widely distributed in mammalian tissues and is responsible for the production of indolamines (serotonin, melatonin), and catecholamines (dopamine, adrenaline, and noradrenaline) [10]. The enzyme AADC is found in serum of various animals such as guinea pig, rat, monkey, mice and humans. Catecholaminergic and serotonergic cells such as adrenal glands, brain, and other tissues as liver and kidney are rich in this enzyme [11].

From a biological point of view the halogen derivatives of tryptamine are not important for metabolism, nevertheless, in many molecules of biological importance hydrogen can be easy replaced by a halogen, without much changing the activity of these species. The introducing of ^18^F, ^79^Br, ^81^Br, and ^131^I in the place of hydrogen atom is widely used for production of radiopharmaceuticals for nuclear medicine and positron emission tomography (PET) [12]. For nuclear medicine it is essential to investigate the metabolism of halogen substituted derivatives of biologically active compounds. Therefore, ^18^F-tryptamine as the potential radiopharmaceuticals [13], is a promising agent for monitoring of abnormal brain states occurring in Parkinson and Alzheimer diseases and schizophrenia, should be a subject of such research.

The studies on the inactive compounds allow to determine the involving of halogenated tryptamines in metabolic reactions. The object of our further study was an investigation whether enzyme AADC effectively catalyzes the oxidative deamination of halogenated derivatives of tryptamine presented in Fig. 1. For study some intrinsic details of mechanism of enzymatic oxidative deamination of halogenated tryptamine and its halogenated derivatives we are going to use the kinetic (KIE) and solvent isotope effects (SIE) methods [14–16].

**Fig. 2:**
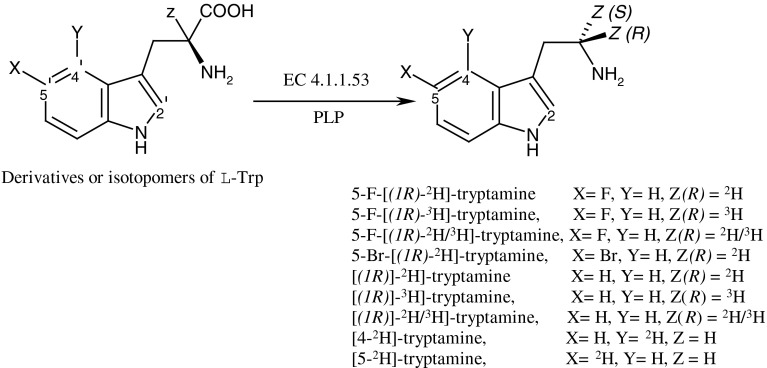
Enzymatic method of synthesis of isotopologues of tryptamine and its derivatives catalyzed by l-phenylalanine decarboxylase (EC 4.1.1.53)

In this work we presented the enzymatic methods of decarboxylation of l-Trp and its halogenated derivatives for producing tryptamine and its halogen derivatives specifically labeled with deuterium and tritium needed to KIE and SIE studies. The decarboxylation carried out in deuteriated or tritiated incubation media was used to synthesis of isotopomers labeled in the aliphatic chain, i.e., [(*1R*)-^2^H]-, [(*1R*)-^3^H]-, [(*1R*)-^2^H/^3^H]-, 5-F-[(*1R*)-^2^H]-, 5-F-[(*1R*)-^3^H], 5-F-[(*1R*)-^2^H/^3^H]-, 5-Br-[(*1R*)-^2^H]-tryptamine, and indole ring labeled ones, i.e. [5-^2^H]-, [4-^2^H]-tryptamine (Fig. 2).

## Experimental

### Materials

Deuteriated water (99.9 % ^2^H), 83 % ^2^H_3_PO_4_/^2^H_2_O and 30 % KO^2^H/^2^H_2_O were purchased from Polatom (Poland). Tritiated water (5 Ci/mL) was from Pharmaceutical Inc., Irvine, CA, USA. The enzyme: l-phenylalanine decarboxylase (*EC 4.1.1.53*) from *Streptococcus faecalis*, and coenzyme PLP (pyridoxal-5′-phosphate) were from Sigma-Aldrich. The chemicals, needed for trial experiments and the enzymatic synthesis, such as l-tryptophan, 5′-fluoro-l-tryptophan, 5′-bromo-l-tryptophan and all other chemicals were from Sigma-Aldrich. TLC plates (DC-Alufolien Kieselgel 60) and silica gel for column chromatography (Kieselgel 60, 0.063–0.200 mm) were from Merck. Liquid scintillation cocktail for aqueous samples was from Ratiszint eco plus (Germany).

### Methods

The ^1^H NMR spectra were recorded in ^2^H_2_O using tetramethylsilane, TMS, as internal standard on a Varian 500 MHz Unity–plus spectrometer. TLC was used for identification of substrates and products in the samples taken in the course of enzymatic reactions. As a developing solvent the water solution of acetonitrile was used (acetonitrile:water; 4:1, v/v). Radioactivity of samples were measured using a liquid scintillation counter Perkin Elmer Tri-Carb 2910TR.

### Synthesis

#### Synthesis of [(1R)-^2^H]-tryptamine, **1**

1. The isotopologue [(*1R*)-^2^H]-tryptamine was obtained by one step reaction by enzymatic decarboxylation of l-tryptophan in fully deuteriated incubation medium. The reaction was carried out in an encapped vial with 50 mg (245 μmol) l-Trp sample dissolved in 15 mL of deuteriated 50 mM Tris–DCl buffer (pD 5.9) to which 4 mg (16 μmol) of PLP and 50 mg (1 U) of enzyme l-phenylalanine decarboxylase (*EC 4.1.1.53*) were added. The reaction mixture was incubated at 37 °C for 2 days and progress of decarboxylation was monitored by TLC. The reaction was quenched by adjusting incubation medium to pH >12 with 30 % KO^2^H/^2^H_2_O. The enzyme was removed by centrifugation, the volume of post-reaction mixture was reduced to *c. a*. 2 mL by evaporation under reduced pressure at 40 °C, loaded on silica gel column (100 × 10 mm), **1** was eluted with acetonitrile solution (CH_3_CN:H_2_O; 5:1, v/v), and collected as 1.5 mL fractions. The presence of **1** in the collected fractions was checked by TLC. The fractions containing **1** were combined and evaporated under reduced pressure at 40 °C and dried under vacuum. As a result 31.7 mg (196 μmol) sample of [(*1R*)-^2^H]-tryptamine was obtained with 80 % chem. yield. The extent of deuterium incorporation (near 100 %) at the (*1R*) position of tryptamine was determined by ^1^H NMR.

#### Synthesis of [(1R-)^3^H]-tryptamine, **2**

2. The **2** was obtained by decarboxylation of l-Trp carried out similarly as in case of **1** using ten times reduced amounts of reagents [1 mL of 50 mM Tris–HCl buffer (pH 5.5), 5 mg (24.5 μmol) of l-Trp, 0.8 mg (3.2 μmol) of PLP, and 5 mg (0.05 U) of l-phenylalanine decarboxylase]. To this reaction mixture 200 μL of tritiated water (total activity of 5.7 GBq) was added. After 2 days the reaction was quenched by changing pH >12 by adding 30 % KOH. The enzyme was centrifuged off and the mixture was lyophilized three times to remove the excess of tritiated water. The residue was dissolved in 200 μL of water and loaded on to silica gel column (100 × 10 mm). Product **2** was eluted with acetonitrile solution (CH_3_CN: H_2_O; 5:1, v/v) and collected as 1.5 mL fractions. From each fraction 10 μL samples was taken for radioassay. The fractions containing radioactive **2** were combined, lyophilized and dried under vacuum. As a results a sample of 3.1 mg (19.1 μmol) of **2** (78 % chem. yield) was obtained with a total activity of 3.7 × 10^4^ Bq (specific activity of 1.49 MBq/mmol).

#### Synthesis of [(1R)-^2^H/^3^H]-tryptamine, **3**

3. This isotopomer, doubly labeled with deuterium and tritium was obtained using the same procedure and amounts of reagents as in the case of **2** [(*1R*)-^3^H]-tryptamine. The decarboxylation of 5 mg (24.5 μmol) l-Trp was carried out in fully deuteriated 50 mM Tris–DCl buffer (pD 5.9) to which 200 μL of ^2^H^3^HO with total activity 5.7 GB was added. As a result 3 mg (18.6 μmol) of doubly labeled [(*1R*)-^2^H/^3^H]-tryptamine was obtained with a total activity of 3 kBq (specific activity of 1.28 × 10^5^ Bq/mmol) and 76 % chem. yield.

#### Synthesis of 5-Br-[(1R)-^2^H]- tryptamine, **4**

4. The sample of 5′-bromo-d, l-Trp (50 mg, 176.5 μmol) was dissolved in 11 mL of deuteriated 50 mM Tris–DCl buffer (pD 5.9) to which 2 mg (8 μmol) of PLP and 26.5 mg (1 U) l-phenylalanine decarboxylase were added. The reaction mixture was incubated at 37 °C for 2 days. The separation and purification of product were the same as in case of isotopomer **1**. Finally, a 7.1 mg (29.7 μmol) sample of **4** was obtained (14.2 % chem. yield). The extent of deuterium incorporation (near 100 %) at the (*1R*) position of **4** was determined by ^1^H NMR spectrum.

#### Synthesis of 5-F-[(1R)-^2^H]-tryptamine, **5**

5. The experimental procedure was similar as in the case of **1**. The sample of 5′-fluoro-l-Trp (25 mg, 112 μmol) was dissolved in 7 mL of deuteriated 50 mM Tris–DCl buffer (pD 5.9) to which 1 mg (4 μmol) of PLP and 26.5 mg (1 U) of l-phenylalanine decarboxylase were added. As a result a 18 mg (101 μmol) sample of **5** was obtained with 90 % chem. yield. The extent of deuterium incorporation (near 100 %) at the (*1R*) position of **5** was determined by ^1^H NMR spectrum.

#### Synthesis of 5-F-[(1R)-^3^H]-tryptamine, **6**

6. The 5.8 mg (26.1 μmol) sample of [5′-F]-l-Trp was dissolved in 1.2 mL of 50 mM Tris–HCl buffer (pH 5.5) to which 0.6 mg (2.43 μmol) of PLP and 5.2 mg (1 U) of enzyme l-phenylalanine decarboxylase were added. To this incubation medium 300 μL of tritiated water (total activity of 8.6 GBq) was introduced. The method of separation and purification of product was the same as in the case of **2**. As the results 3.9 mg (21.8 μmol) of **6** was obtained (84 % chem. yield) with total activity 3.1 × 10^4^ Bq (specific activity of 1.24 MBq/mmol).

#### Synthesis of 5-F-[(1R)-^2^H/^3^H]-tryptamine, **7**

7. This product was obtained in the same manner as described in the case of **3.** The sample of 5.5 mg (24.7 μmol) [5′-F]-l-Trp was dissolved in 1.4 mL of 50 mM Tris–DCl buffer, pD 5.9, to which 0.6 mg (2.43 μmol) of PLP, 5.2 mg (1 U) of l-phenylalanine decarboxylase, and 350 μL of ^2^H^3^HO (total activity of 10 GBq) were added. Finally, a 3.6 mg (20.3 μmol) sample of **7** was afforded (82 % chem. yield) with total activity 8.7 × 10^4^ Bq (specific activity of 3.56 MBq/mmol).

#### Synthesis of [4-^2^H]-tryptamine, **8**

8. The sample of 10 mg (48.8 μmol) of [4′-^2^H]-l-Trp, obtained as described earlier [17] was dissolved in 1 mL 50 mM Tris–HCl buffer (pH 5.5) to which 2 mg (8 μmol) of PLP and 10 mg (1 U) of l-phenylalanine decarboxylase were added. The experimental protocol of synthesis, separation, and purification of **8** was the same as described for derivative **1**. As a result a 6.4 mg (39.8 μmol) sample of **8** was obtained with 82 % chem. yield and 73 % of deuterium enrichment at the 4′-indole ring position determined by ^1^H NMR.

#### Synthesis of [5-^2^H]-tryptamine, **9**

9. The compound **9** was obtained by the same protocol as in case of **8** using as a substrate [5′-^2^H]-l-Trp synthesized according modified procedures described earlier [18]. The sample of 3 mg (14.6 μmol) of [5′-^2^H]-l-Trp (100 % ^2^H enrichment) was dissolved in 50 mM Tris–HCl buffer pH 5.5 to which 0.6 mg (2.43 μmol) of PLP and 5.2 mg (1 U) l-phenylalanine decarboxylase were added. As a result a 1.8 mg (11.4 μmol) of **9** was obtained (78 % chem. yield) with 100 % of deuterium enrichment.

## Results and discussion

**Fig. 3:**
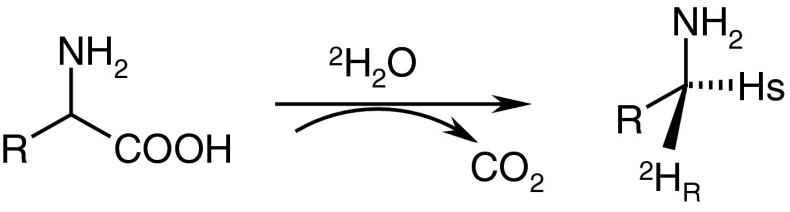
The stereochemistry of the enzymatic decarboxylation of α-amino acids

Generally, all nine isotopomers of tryptamine and its halogen derivatives were synthesized in course of enzymatic decarboxylation, Fig. 1, of appropriate derivatives of l-tryptophan. For this reaction instead of the enzyme aromatic l-ADDC (*EC 4.1.1.28*) l-phenylalanine decarboxylase (*EC 4.1.1.53*) from *Streptococcus faecalis* [14] (having the similar properties as ADDC) was used. Previous studies [19, 20] has strongly documented that enzymatic decarboxylation of α-l-amino acids undergoes together with the replacement of carboxyl group by solvent proton (deuteron/triton) with retention of configuration at the α-carbon (Fig. 3).

Therefore, the enzymatic decarboxylation of appropriated derivative of l-Trp carried out in fully deuteriated or tritiated medium produces corresponding tryptamine labeled with deuterium or tritium in configuration (*1R*). Introduction of one deuterium (or tritium) atom from incubation medium to the newly generated tryptamine and preserving the configuration at the carbon atom in the α-position leads to obtain *R*-isotopologues of tryptamines [19–22].

The tritium labeled compounds **2** and **6**, i.e., [(*1R*)-^3^H]-, and 5-F-[(*1R*)-^3^H]- tryptamine were synthesized by enzymatic decarboxylation of l-Trp and [5′-F]-l-Trp carried out in tritiated (^1^H^3^HO) Tris–HCl buffered medium, pH 5.5 (at this pH value the enzyme l-phenylalanine decarboxylase shows the maximum activity). The deuteriated compounds **1**, **4**, and **5**, i.e., [(*1R*)-^2^H]-, 5-Br-[(*1R*)-^2^H]- and 5-F-[(*1R*)-^2^H]-tryptamine were obtained by decarboxylation of l-Trp, [5′-Br]-d, l-Trp, and [5′-F]-l-Trp, respectively, in fully deuteriated Tris–DCl buffer, pD 5.9. The compounds **3** and **7** doubly labeled with deuterium and tritium, i.e., [(*1R*)-^2^H/^3^H]- and 5-F-[(*1R*)-^2^H/^3^H]-tryptamine were synthesized in fully deuteriated Tris–DCl buffer, pD 5.9, to which tritiated water was added. In the case when the synthesis was carried out in fully deuteriated incubation medium its pD was corrected to 5.9 value due to higher pK (D_2_O) [23]. In the case of deuteriated media all substrates for decarboxylation reaction were dissolved in almost entirely deuteriated 50 mM Tris–DCl buffer (394 mg, 2.5 mmol) of Tris–HCl were dissolved in 50 mL of D_2_O; and calculated fraction of H^+^/D^+^ ions in this way prepared incubation medium was equal to 0.0005).

The compound **8**, i.e., [4-^2^H]-tryptamine, labeled with deuterium in the 4-position of indole ring was synthesized by decarboxylation of [4′-^2^H]-l-Trp which was obtained by ^1^H/^2^H isotope exchange according the procedure described earlier [24, 25]. The exchange was catalyzed with UV light produced by a 250 W mercury lamp. The sample of l-Trp dissolved in ^2^H_2_O was irradiated in a sealed and outgassed glass ampoule. The ^1^H NMR spectrum showed that deuterium enrichment at the 4′ position of l-Trp was around 73 %.

The indole ring labeled compound **9**, i.e. [5-^2^H]-tryptamine was obtained by decarboxylation of [5′-^2^H]-l-Trp synthesized according earlier described procedures [18, 25]. This intermediate [5′-^2^H]-l-Trp was obtained in two step synthesis: first 5-bromoindole was reduced by sodium borodeuteride, NaB^2^H_4_, to[5-^2^H]-indole [25], which in next step, catalyzed by enzyme tryptophanase (l-tryptophan indole-lyase *EC 4.1.99.1*), was coupled with *S*-methyl-l-cysteine giving [5′-^2^H]-l-Trp [18] (Near 100 % deuterium incorporation in the 5′-position by ^1^H NMR spectrum). The enzymatic decarboxylations of [4′-^2^H]-, and [5′-^2^H]-l-Trp, leading to **8** and **9** were carried out in 50 mM Tris–HCl buffer, pH 5.5.

The derivative **4**, i.e. 5-Br-[(*1R*)-^2^H]-tryptamine was obtained with relatively low chemical yield (about 15 %) as for decarboxylation reaction. It is possible that, this enzyme is less effective catalyst for decarboxylation of [5′-Br]-l-Trp with such large ring substituent as bromine. For this reason, we made no attempts to synthesize its isotopologue, i.e. tritiated 5-Br-[(*1R*)-^3^H]-tryptamine.

The extent of deuterium incorporation in the (*1R*)-position of tryptamines was determined by measuring the signal integration derived from the α-protons in ^1^H NMR spectrum. In the same manner the deuterium enrichment in the 4′- and 5′-positions of intermediates [4′-^2^H]-, and [5′-^2^H]-l-Trp was identified.

## References

[1] Kema Ido P, De Vries Elisabeth GE, Muskiet Frits AJ (2000). Clinical chemistry of serotonin and metabolites. J Chromatogr B.

[2] Itoh MT, Ishizuka B, Kuribayashi Y, Amemiya A, Sumi Y (1999). Melatonin, its precursors, and synthesizing enzyme activities in the human ovary. Mol Reprod.

[3] Jacob MS, Presti DE (2005). Endogenous psychoactive tryptamines reconsidered: an anxiolytic role for dimethyltryptamine. Med Hypoth.

[4] Brandt SD, Martins CPB (2010). Analytical methods for psychoactive *N*,*N*-dialkylated tryptamines. TrAC.

[5] Maximino C (2012). Serotonin and anxiety, serotonin in the nervous system of vertebrates.

[6] Hocck DR, Floss HG (1981). Preparation of stereospecifically α- and β-tritiated tryptamine and the stereochemistry of aromatic l-amino acid decarboxylase. J Nat Prod.

[7] Slominski A, Semak I, Pisarchik A, Sweatman T, Szczesniewski A, Wortsman J (2002). Conversion of l-tryptophan to serotonin and melatonin in human melanoma cells. FEBS Lett.

[8] Lovenberg W, Weissbach H, Underfriend S (1962). Aromatic l-amino acid decarboxylase. J Biol Chem.

[9] Gale EF (1945). Studies on bacterial amino-acid decarboxylases. Biochem J.

[10] Nagai T, Hamada M, Kai N, Tanoue Y, Nagayama F (1995). Purification and properties of aromatic l-amino acid decarboxylase from liver of skipjack tuna. Comp Biochem Physiol.

[11] Nishigaki I, Ichinose H, Tamai K, Nagatsu T (1988). Purification of aromatic l-amino acid decarboxylase from bovine brain with a monoclonal antibody. Biochem J.

[12] Bomanji JB, Costa DC, Ell PJ (2001). Clinical role of positron emission tomography in oncology. Lancet Oncol.

[13] Pacak K, Eisenhofer G, Carrasquillo JA, Chen CC, Sheng-Ting Li, Goldstein DS (2001). 6-[^18^F] fluorodopamine positron emission tomographic (PET) scanning for diagnostic localization of pheochromocytoma. Hypertension.

[14] Wigley LJ, Mantle PG, Perry DA (2006). Natural and directed biosynthesis of communesin alkaloids. Phytochemistry.

[15] Huskey WP, Cook PF (1991). Origins and interpretations of heavy-atom isotope effects. Enzyme mechanism from isotope effects.

[16] Schowen RI (1972). Mechanistic deductions from solvent isotope effects. Prog Phys Org Chem.

[17] Saito I, Sugiyama H, Yamamoto A, Muramatsu S, Matsuura T (1984). Photochemical hydrogen-deuterium exchange reaction of tryptophan. The role in nonradioative decay of singlet tryptophan. J Am Chem Soc.

[18] Kiick DM, Phillips RS (1988). Mechanistic deductions from multiple kinetic and solvent deuterium isotope effect and pH studies of pyridoxal phosphate dependent carbon–carbon lyases: *Escherichia coli* tryptophan indole-lyase. Biochemistry.

[19] Belleau B, Burba J (1960). The stereochemistry of the enzymic decarboxylation of amino acids. JACS.

[20] Battersby R, Scott A, Staunton J (1990). Studies of enzyme-mediated reactions. Stereochemical course of the formation of 5-hydroxytryptamine (serotonin) by decarboxylation of (2S)-5-hydroxytryptophan with the aromatic l-amino acid decarboxylase (EC 4.1.1.28) from hog kidney. Tetrahedron.

[21] Vederas JC, Reingold ID, Sellers HW (1979). Stereospecificity of sodium borohydride reduction of tyrosine decarboxylase from *streptococcus faecalis*. J Biol Chem.

[22] Chang GW, Snell EE (1968). Histidine decarboxylase of *Lactobacillus* 30a. II. Purification, substrate specificity, and stereospecificity. Biochemistry.

[23] Gary R, Bates RG, Robinson RA (1964). Second dissociation constant of deuteriophosphoric acid in deuterium oxide from 5 to 50 °C: standardization of pD scale. J Phys Chem.

[24] Winnicka E, Kańska M (2009). Synthesis of l-tryptophan labeled with hydrogen isotopes in the indole ring. J Radioanal Nucl Chem.

[25] Bosin TR, Raymond MG, Buckpitt AR (1973). A site-specific method for deuteration: reduction of aryl halides with sodium borodeuteride and palladium chloride. Tetrahedron Lett.

